# Prolonged preoperative treatment of acromegaly with Somatostatin analogs may improve surgical outcome in patients with invasive pituitary macroadenoma (Knosp grades 1–3): a retrospective cohort study conducted at a single center

**DOI:** 10.1186/s12902-017-0205-3

**Published:** 2017-09-06

**Authors:** Lian Duan, Huijuan Zhu, Bing Xing, Feng Gu

**Affiliations:** 1 Key Laboratory of Endocrinology, Ministry of Health; Department of Endocrinology, Peking Union Medical College Hospital, Peking Union Medical College and Chinese Academy of Medical Sciences, Beijing, 100730 China; 2 Department of Neurosurgery, Peking Union Medical College Hospital, Peking Union Medical College and Chinese Academy of Medical Sciences, Beijing, 100730 China

**Keywords:** Acromegaly, Preoperative treatment, Somatostatin analogs, Invasive macroadenoma, Remission rate

## Abstract

**Background:**

This study aimed to investigate preoperative somatostatin analogs (SSAs) treatment on the surgical outcome in patients with acromegaly.

**Methods:**

An analysis of 358 patients with acromegaly was conducted. The preoperative medical therapy group (81 patients) received SSA treatment for at least 3 months prior to surgery, while the primary surgery group (277 patients) underwent transsphenoidal surgery directly. Follow-up duration was ≥3 months. Tumor invasion was evaluated by magnetic resonance imaging (MRI) and classified according to the Knosp grading system.

**Results:**

Most patients were diagnosed with macroadenoma. Among all patients (Knosp grades 0–4), preoperative SSA therapy did not significantly improve the curative effect of surgery, according to the levels of growth hormone (GH) and/or insulin-like growth factor 1 (IGF-1) markers. In patients with macroadenoma (Knosp grades 1–3), the remission rates were significantly higher in the SSA group compared to the surgery group when considering GH (56.4% vs. 37.3%, *P* = 0.048) and IGF-1 (43.2% vs. 17.6%, *P* = 0.004). In the preoperative medical therapy group, long-term use of SSAs (>6 months) led to higher remission rates (GH, 72.2% vs. 51.0%; and IGF-1, 61.1% vs. 29.8%; *P* = 0.12 and 0.02, respectively].

**Conclusions:**

The long-term preoperative SSAs treatment may improve the surgical curative rate in acromegalic patients with invasive macroadenomas (Knosp grades 1–3).

**Electronic supplementary material:**

The online version of this article (10.1186/s12902-017-0205-3) contains supplementary material, which is available to authorized users.

## Background

Acromegaly is a chronic disease that is caused, in the great majority of cases, by growth hormone (GH)-producing pituitary tumors. Hypersecretion of GH leads to excessive production of insulin-like growth factor 1 (IGF-1), leading to a multisystem disease characterized by somatic overgrowth, multiple comorbidities, premature mortality, and physical disfigurement. The main complications of untreated acromegaly are cardiovascular disease and malignancy, leading to high mortality [1, 2]. A multidisciplinary approach is critical for the management of this disorder and may decrease the incidence of these complications [1–3].

Transsphenoidal surgery (TSS) is the accepted first-line treatment for acromegaly in most patients. Surgical results are dependent on the preoperative serum GH and IGF-1 levels, extent of tumor invasion, and the skill of the neurosurgeon. Overall surgical remission rates are estimated to 75–90% for microadenomas and 40–60% for non-invasive macroadenomas, but this rate falls to 10–20% for invasive macroadenomas [4].

Consequently, additional therapy is required in order to reduce the symptoms associated with macroadenomas and to control the GH and IGF-1 levels following initial TSS. Medical therapeutic interventions such as the use of dopamine agonists and somatostatin analogs (SSAs) as adjuvant therapy are associated with favorable outcomes [5, 6]. SSAs are commonly used to control symptoms and hormonal hypersecretion in patients after unsuccessful primary surgery. SSA treatment can be extended for a long-term period and in combination with radiotherapy.

Primary SSAs therapy is used in patients with low probability of surgical remission and when TSS is not feasible. Primary SSAs therapy causes a decline in the hormone levels in most patients, but the individual patient response variation is high. About two thirds of SSA-naïve patients experience significant tumor shrinkage during SSA treatment [5–8]. Preoperative SSA treatment has been shown to result in improved symptomatic and metabolic control (particularly glucose tolerance and blood pressure), reduced soft tissue swelling, reduced tumor size/grade and, occasionally, altered and/or improved tumor consistency [9].

The guidelines from the European Society of Endocrinology and the American Association of Clinical Endocrinologists in 2014 recommended that preoperative SSA treatment should not be used routinely to improve the postoperative biochemical remission rate of acromegaly [10, 11]. Although preoperative SSA treatment alleviates the symptoms and reduces the risk of complications during surgery, this approach remains controversial [12]. To date, four prospective cohort studies suggest that preoperative SSA treatment may increase the recovery rate following surgery for macroadenoma [13–16]. Nevertheless, the postoperative remission rates of the patients with macroadenoma following direct surgery (without SSA treatment) ranged between 10% and 20% in these four studies [13–16].

These studies [13–16] suggested that preoperative SSA treatment could be used to increase the surgical recovery rate of patients with macroadenoma, including those with invasive disease. In addition, the duration of SSA treatment in the literature ranges from 3 to 6 months, while the efficacy of prolonged preoperative SSA treatment (˃6 months) on surgical recovery remains unclear. Therefore, the aim of the present retrospective study was to investigate whether the course and preoperative administration of SSAs improve the outcome of surgery for acromegaly.

## Methods

### Study design

The present study was a retrospective cohort analysis of patients who underwent the resection of GH-secreting pituitary tumors at the Peking Union Medical College Hospital between 2009 and 2014. This study was approved by the Ethics Committee of the Chinese Academy of Medical Sciences and Peking Union Medical College Hospital. The need for individual consent was waived by the committee due to the retrospective nature of the study.

### Patients

The inclusion criteria were: 1) 18–80 years of age; 2) confirmed diagnosis of acromegaly based on biochemical tests (fasting GH >2.5 μg/L, lowest GH >1 μg/L in glucose GH inhibition tests, and IGF-1 levels higher than the normal range for age- and sex-matched healthy subjects); 3) confirmed pituitary tumor in the sellar area as determined by magnetic resonance imaging (MRI) or CT scan; and 4) initial diagnosis of acromegaly and pituitary tumor resection at our hospital.

The exclusion criteria were: 1) prior surgical treatment or radiotherapy for a pituitary tumor; 2) the duration of preoperative SSA treatment was <3 months; 3) the interval between the preoperative SSA treatment and surgery was >3 months; 4) treatment with dopamine receptor agonists before surgery; 5) postoperative follow-up <3 months; 6) secondary surgery or radiotherapy within 3 months following the operation; and 7) treatment with SSAs or dopamine receptor agonists within 3 months following the operation.

The selection of the treatment approach was decided by the physicians and the patients, based on the Chinese diagnostic and treatment guidelines for acromegaly [17, 18] and on the patients’ capacity to pay for SSAs (medical insurance or not). The patients were divided into two groups: 1) the preoperative medical therapy group (received preoperative SSAs treatment); and 2) the primary surgery group (underwent direct surgical treatment, without SSA therapy before surgery).

### Intervention

All the operations were conducted by the surgeons from the Neurosurgery Department of the Peking Union Medical College Hospital. The patients in the preoperative medical therapy group underwent preoperative SSA treatment using octreotide long-acting repeatable (LAR) (20 mg, every 28 days; the dosage was increased to 40 mg, every 28 days when GH and IGF-1 were not adequately controlled) or somatuline (40 mg, every 14 days; dose interval was reduced to 10 days when the biochemical target index could not be achieved). The dose was adapted according to the clinical symptoms of the patients and the remission rates [19, 20]. Surgery was conducted via the endonasal transsphenoidal approach for the treatment of pituitary tumors [4, 10, 11].

### Indicators and measurements

The patients were followed-up for at least 3 months following surgery. Postoperative remission was determined according to the Chinese biochemical remission criteria for acromegaly (2013) [17, 18]: random serum GH <2.5 μg/L, glucose GH inhibition test that exhibited GH levels lower than 1 μg/L, and/or IGF-1 levels that were decreased to normal levels according to the age and the sex of the patients. An oral glucose tolerance test (OGTT) has been proposed as an alternative method to assess remission in acromegaly [21, 22], but several studies have supported the use of GH and IGF-1 biomarkers in assessing remission in acromegaly [23–26]. The disease was evaluated at diagnosis, at 3 months following surgery, and at the last visit (≥3 months following surgery).

GH and IGF-1 levels were measured from fasting venous blood samples using a chemiluminescence method (IMMULITE2000 Growth Hormone (hGH) and IMMULITE2000 IGF-I, Siemens Healthcare Diagnostics, USA). For the glucose GH inhibition test, the patients were asked to ingest 75 g glucose in the morning without food, and the venous blood was obtained prior to and at 30, 60, 90, and 120 min following glucose ingestion.

### Tumor invasiveness classification by MRI

A total of 202 patients with macroadenoma (67 in the preoperative medical therapy group and 135 in the primary surgery group) underwent sellar MRI scans at the Peking Union Medical College Hospital during at diagnosis. None of the patients had received any treatment (including drug therapy or surgery). Tumor invasion was evaluated by the doctors in the Radiology Department according to the Knosp criteria. The patients with invasive macroadenoma were grouped as the Knosp 1–3 and Knosp 4 subgroups. This selection was carried out because of the limited number of Knosp 4 patients, while both Knosp 1 and 2 tumors demonstrate considerably low percentage of invasion [27, 28].

The Knosp grading system comprises five categories [19, 20]: (0) no invasion with all of the lesion medial to the cavernous carotid artery (CCA); (1) invasion extending to, but not beyond, the medial aspect of the CCA; (2) invasion extending to, but not beyond, the lateral aspect of the CCA; (3) invasion past the lateral aspect of the CCA, but not completely filling the cavernous sinus (CS); and (4) completely filling the CS both medial and lateral to the CCA. Invasive macroadenoma was defined as macroadenoma with CS extension (Knosp ≥1).

### Statistical analysis

Statistical analysis was carried out using the SPSS 16.0 software package (IBM, Armonk, NY, USA). Continuous data were expressed as mean ± standard deviation (SD). The chi-square test and Fisher’s exact test were used to compare remission rates. Two-sided *P*-values <0.05 were considered statistically significant.

## Results

### Characteristics of the patients

**Fig. 1:**
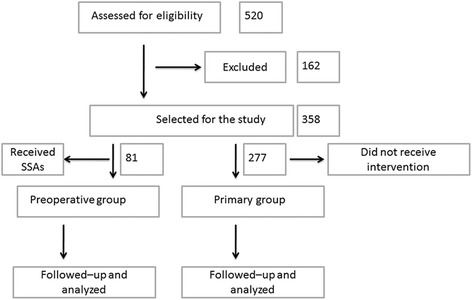
Flow diagram of patient recruitment and selection for the study

A total of 520 patients were screened and 358 patients were eligible and met the study criteria (Fig. 1). Among these patients, the male (*n* = 166) to female (*n* = 192) ratio was 1:1.16, the mean age at disease onset was 34.7 years (range, 15–70 years), and the mean age at disease diagnosis was 40.3 years (range, 18–71 years). The Knosp grade system was used as an alternative stratification method.

The preoperative medical therapy group included 81 patients, 10 of which were diagnosed with microadenoma (adenoma diameter ≤ 1 cm), while 71 were diagnosed with macroadenoma (adenoma diameter > 1 cm). The preoperative drug treatment lasted 3–36 months, and the median treatment duration was 4 months. Only one patient had taken drug therapy for 36 months prior to surgery, with octreotide LAR 20 mg.

A total of 29 out of 277 patients who underwent initial surgery were diagnosed with microadenoma while the remaining 248 were diagnosed with macroadenoma. The patient characteristics at baseline are shown in Table 1.

**Table 1:** Patient characteristics at baseline

	Preoperative medical therapy (*n* = 81)	Primary surgery (*n* = 277)	*P*
M/F	41/40	125/152	0.38
Age of onset (y)	34.8 ± 11.4	34.6 ± 11.7	0.86
Age of diagnosis (y)	40.3 ± 11.9	40.1 ± 12.1	0.89
Mean basal GH (μg/L)	44.47 ± 16.13	48.17 ± 17.52	0.68
Mean IGF-1 (μg/L)	841.27 ± 313.26	889.50 ± 306.21	0.31
Microadenoma (%)	10 (12.3)	29 (10.5)	0.63
Macroadenoma (%)	71 (87.7)	248 (89.5)	0.63
^a^Tumor invasion (Knosp)	67	135	0.161
Knosp 0 (%)	21 (31.3)	49 (36.3)	
Knosp 1 (%)	6 (9.0)	12 (8.9)	
Knosp 2 (%)	19 (28.4)	42 (31.1)	
Knosp 3 (%)	14 (20.9)	29 (21.5)	
Knosp 4 (%)	7 (10.4)	3 (2.2)	

^a^Classified according to magnetic resonance imaging performed at Peking Union Medical College Hospital (China)

Age at disease onset, disease duration, baseline biochemical parameters, and percentage of patients with macroadenoma were not significantly different between the two groups.

Among the patients with macroadenoma, 67 were in the preoperative medical therapy group, among which 21, 6, 19, 14, and 7 exhibited Knosp disease of grade 0, 1, 2, 3, and 4, respectively. This corresponded to an estimated percentage of 31.3%, 9.0%, 28.4%, 20.9%, and 10.4%, respectively. A total of 135 of the patients with macroadenoma were included in the primary surgery group. Among those patients 49, 12, 42, 29, and 3 corresponding to 36.3%, 8.9%, 31.1%, 21.5%, and 2.2%, respectively, exhibited disease Knosp grade 0, 1, 2, 3, and 4, respectively (Table 1).

A total of 117 out of 319 patients with invasive macroadenoma (36.7%) underwent MRI scans at other hospitals, with four patients (5.6%) in the preoperative medical therapy group and 113 patients (45.6%) in the primary surgery group. Consequently, the latter patients did not undergo further MRI evaluation or Knosp grading.

### Remission rate after surgery

The patients were followed-up for at least 3 months following surgery (3–53 months, mean follow-up time: 11.1 ± 10.9 months). GH levels were controlled in 46 out of 81 (56.8%) of patients treated with SSAs compared with 143 out of 277 (51.6%) patients who underwent surgery directly (*P* = 0.41). In contrast to the latter observation, 32 out of 79 (40.5%) of SSA- pretreated patients exhibited normalized IGF-1 level compared with 83 of 255 (32.6%) patients who underwent surgery (*P* = 0.19). This classification was conducted according to the biochemical remission criteria mentioned in the Chinese guideline.

In patients with microadenomas, the remission rate of GH in the preoperative medical therapy group was significantly higher compared with that of the primary surgery group (100% vs. 62.1%, *P* = 0.037), while the remission rate of IGF-1 was higher in the preoperative medical therapy group (60% vs. 35.7%, *P* = 0.267).

In patients with macroadenomas, the preoperative medical therapy did not improve the curative effect of surgery and there was no significant difference in the GH (50.7% vs. 50.4%, *P* = 0.96) or IGF-1 (37.7% vs. 32.2%, *P* = 0.39) remission rates between the two groups (Table 2).

**Table 2:** Surgical remission rates of patients with acromegaly

		Preoperative medical therapy	Primary surgery	*P*
Remission rate for GH	Total (%)	46/81 (56.8)	143/277 (51.6)	0.41
	Microadenoma (%)	10/10 (100)	18/29 (62.1)	0.037
	Macroadenoma (%)	36/71 (50.7)	125/248 (50.4)	0.96
Remission rate for IGF-1	Total (%)	32/79 (40.5)	83/255 (32.6)	0.19
	Microadenoma (%)	6/10 (60)	10/28 (35.7)	0.267
	Macroadenoma (%)	26/69 (37.7)	73/227 (32.2)	0.39

### Remission rate of macroadenoma according to Knosp grade

CS invasion is considered one of the most significant preoperative predictor of remission in the surgical treatment of pituitary GH adenomas. The remission rate for GH and IGF-1 decreased significantly with higher Knosp grade in both groups. The biochemical remission rates in the primary surgery group did not reveal a significant difference compared with those of the preoperative medical therapy group in patients with Knosp grade 0 tumors (Table 3), but in patients with invasive macroadenoma (Knosp grade 1–3), the GH and IGF-1 remission rates were significantly higher in the preoperative medical therapy group compared with those in the primary surgery group (Table 3). No significant differences between the two groups were noted for Knosp grade 4 macroadenomas (Table 3). As a result, the patients with invasive macroadenomas (Knosp grade 1–3) may benefit from medical therapy prior to surgery (Table 3).

**Table 3:** Surgical remission rates of patients with macroadenoma according to the Knosp grade

		Preoperative medical therapy	Primary surgery	*P*
Remission rate for GH	Total (%)	38/67 (56.7)	68/135 (50.4)	0.40
	Knosp grade 0 (%)	15/21 (71.4)	37/49 (75.5)	0.72
	Knosp grade 1–3 (%)	22/39 (56.4)	31/83 (37.3)	0.048
	Knosp grade 4 (%)	1/7 (14.3)	0/3 (0)	~1.00
Remission rate for IGF-1^a^	Total (%)	25/65 (38.5)	42/125 (33.6)	0.51
	Knosp grade 0 (%)	8/21 (38.1)	29/48 (60.4)	0.09
	Knosp grade 1–3 (%)	16/37 (43.2)	13/74 (17.6)	0.004
	Knosp grade 4 (%)	1/7 (14.3)	0/3 (0)	~1.00

^a^IGF-1 results were not available for all patients

### Remission rate of macroadenoma according to preoperative SSA therapy duration

The duration of the preoperative SSA therapy in some of the patients was >3–6 months, as recommended in previous studies [12]. To investigate the effects of the duration of the preoperative medical therapy on the surgical remission rate, the patients with macroadenoma were divided into two subgroups: ≤6 months (median time, 3 months) and >6 months (median time, 12 months). Comparison of the latter subgroups with the primary surgery group (Table 3) showed no significant differences in the mean GH and IGF-1 levels at diagnosis, but the IGF-1 remission rates in the patients who received >6 months of preoperative SSA treatment were significantly greater compared with those of the patients who underwent surgery directly and/or those who received ≤6 months of preoperative SSA treatment (Table 4). With regard to the GH, levels no significant difference in remission was noted between the two groups (Table 4). Among the patients with Knosp grade 1–3 disease, a significant difference was noted for the remission rate of the IGF-1 levels between the two groups as regards the Knosp grade 1–3, whereas the differences noted in the cases of Knosp 0 and 4 grades were not statistically significant (Table 4).

**Table 4:** Surgical remission rates in patients with macroadenoma according to preoperative SSA therapy duration

	SSAs ≤6 months (*n* = 49)	SSAs >6 months (*n* = 18)	*P*
Mean basal GH at diagnosis (μg/L)	46.11 ± 16.40	35.96 ± 14.69	0.383
Mean IGF-1 at diagnosis (μg/L)	874.9 ± 309.7	789.3 ± 257.1	0.702
Mean SSAs therapy duration (month)	3.6 ± 0.98	18.1 ± 11.0	<0.001
Remission rate for GH	25/49 (51.0)	13/18 (72.2)	0.121
Knosp grade 0 (%)	10/15 (66.7)	5/6 (83.3)	0.623
Knosp grade 1–3 (%)	15/28 (53.6)	7/11 (63.6)	0.725
Knosp grade 4 (%)	0/6 (0)	1/1 (100)	0.143
Remission rate for IGF-1	14/47 (29.8)	11/18 (61.1)	0.020
Knosp grade 0 (%)	4/15 (26.7)	4/6 (66.7)	0.146
Knosp grade 1–3 (%)	10/26 (38.5)	6/11 (54.5)	0.018
Knosp grade 4 (%)	0/6 (0)	1/1 (100)	0.143

## Discussion

The present study was a retrospective analysis of the effects of preoperative SSA treatment on the surgical curative rate of patients with acromegaly who underwent pituitary tumor resection via the transsphenoidal approach at our hospital from 2009 and 2014. According to the biochemical remission criteria recommended by the Chinese diagnostic and treatment guidelines for acromegaly [17, 18], the postoperative GH (100% vs. 62.1%) and IGF-1 (60% vs. 35.7%) remission rates in the patients with microadenoma who received preoperative SSAs treatment were higher compared with those in the patients who underwent surgery directly. Preoperative treatment did not increase the postoperative biochemical remission rate in the patients with macroadenoma. The subgroup analysis indicated that preoperative SSAs increased the postoperative biochemical remission rate in the patients with macroadenoma of Knosp grades ≥1. In addition, the surgical efficacy and biochemical remission rate were further improved by the administration of preoperative SSA treatment for a time period >6 months.

It has been reported that a majority of patients that present with acromegaly due to GH-secreting tumors exhibit inadequately controlled disease following surgery and that the use of SSAs is encouraged in order to improve the clinical symptoms [21, 29–31]. Although the use of drug therapy in pituitary tumors has traditionally been limited to the adjuvant setting, first-line treatment with SSAs can be used in selected patients, including those with invasive tumors, those at risk of complications associated with anesthesia, those with severe complications of acromegaly, those who refuse surgery, and those who desire to retain intact pituitary function. It has been suggested that tumor removal during surgery is facilitated by SSA pretreatment [27, 28]. Previous studies addressing preoperative SSAs and subsequent surgical cure rates are conflicting [32–37]. In the present study, the preoperative SSA treatment increased the postoperative GH (100% vs. 62.1%) and IGF-1 (60% vs. 35.7%) remission rates in patients with microadenoma, although the results were significantly different for the GH and not for the IGF-1 levels. In addition, the preoperative medical treatment did not increase the overall surgical remission rates in patients with macroadenoma.

The success rates of TSS decline substantially in patients harboring large and invasive tumors and alternative therapeutic modalities are required when TSS fails [21, 29]. In one study, male sex and parasellar extension (especially CS invasion) were the most powerful predictors of persistent disease [30, 31]. Knosp grade (0–2) and GH levels (<45 ng/mL) have been shown to correlate with surgery remission in patients with GH-secreting tumors compared with preoperative demographics and tumor characteristics (which did not exhibit an association) [38, 39]. The present study showed that the postoperative GH and IGF-1 remission rates in patients with Knosp grade 0 macroadenoma who underwent surgery directly were 75.5% and 60.4%, respectively, but that the postoperative GH and IGF-1 remission rates decreased markedly with increasing CS invasion. No cases of biochemical remission were noted among the patients with Knosp grade 4 tumors.

The medical treatment with SSAs reduced the GH and IGF-1 levels effectively and decreased the tumor size to some extent. Based on these observations, the involvement of the preoperative SSAs treatment in the increase of the surgical recovery rate in patients with invasive macroadenoma was investigated. The subgroup analysis indicated that in the Knosp grade 0 subgroup, the preoperative SSA treatment did not increase the surgical efficacy. In contrast to this finding, the postoperative GH and IGF-1 remission rates in the patients who received preoperative SSAs treatment and exhibited invasive macroadenoma (Knosp grade ≥ 1) were significantly greater compared with those in the patients who underwent surgery directly. The overall postoperative remission rate among the patients with Knosp grade 4 macroadenoma was very low, possibly due to the limited number of cases included in the present study. As a consequence, this group was not included in the analysis. In the subsequent analysis, the preoperative SSAs treatment significantly increased the surgical curative rates in the remaining patients with invasive macroadenoma (Knosp grades 1–3). The aforementioned findings indicate that the preoperative SSAs treatment provides beneficial effects in invasive macroadenoma patients with tumors that do not progress with complete CS invasion.

A review of published studies evaluating SSAs as first-line therapy has reported that octreotide LAR therapy for a period of 6–24 months conferred significant (20–30%) tumor volume reduction in 73–85% of patients, with an overall mean reduction in tumor volume of 35–68% [40]. In a prospective, multicenter study of 98 patients treated with octreotide LAR at a dose of 10–30 mg every 4 weeks, a reduction in tumor volume exceeding 20% tumor was reported in 63% and 75% of patients following 24 and 48 weeks of treatment, respectively. Furthermore, it has been shown that tumor shrinkage increases with time, with the largest decreases in tumor volume generally occurring in the first year of treatment [41]. To date, the studies that have addressed the effects of preoperative SSA treatment on the surgical outcomes have adopted a 3–6-month regimen. The novelty of the present study is focused on the effects of the preoperative SSAs duration on the postoperative biochemical remission rate, which have not yet been reported. In addition, the patients were divided into two subgroups according to the preoperative SSAs treatment duration (≤6 months and >6 months). In the present study, for IGF-1, the difference between the ≤6 and >6 months groups was significant (61.1% vs. 29.8%). Further subgroup analysis showed that treatment for ≤6 and >6 months had differential effects on IGF-1 in patients with Knosp 1–3 disease (54.5% vs. 38.5%). Considering that the patients with Knosp 1–3 disease and who directly underwent surgery only have a 17.6% remission rate, extension of pre-operative SSA treatment could be especially helpful. On the other hand, no effect of pre-operative treatment duration (≤6 vs. >6 months) was observed for GH despite that differences were observed for pre-operative treatment (regardless of duration) vs. direct surgery for Knosp grade 1–3 subjects.

It is important to highlight that the current study was focused on acromegalic patients and did not examine the possibility of potentially deranged pituitary axis, of co-existing conditions such as central adrenal insufficiency, and of impaired patterns of other hormones including prolactin. The selection of the subjects was based solely on the diagnosis of acromegaly. The diagnosis of acromegaly was confirmed by biochemical tests and MRI or CT scans that indicated the presence of the pituitary tumor in the sellar area. This diagnosis excluded the manifestation of further pituitary-associated hormonal conditions and symptoms.

The present study is limited by its retrospective nature, i.e. selection bias and information bias [42]. The lack of control, the lack of randomization, the single center, and the inclusion of additional clinicopathological parameters during SSA treatment in the statistical analysis of the study are all disadvantages that can be improved in a future study. In addition, the decrease in tumor invasion was not investigated in the patients who received preoperative drug therapy for >6 months. Furthermore, the postoperative GH and IGF-1 levels in some patients were not consistent [42] and the decrease in the IGF-1 levels following the operation was relatively slow, indicating that the IGF-1 remission rate was significantly lower than the GH remission rate in the present study. Nevertheless, similar retrospective analysis of acromegaly patients who received 3–6 months of preoperative SSAs showed that this time duration is optimal [17, 18, 43]. The numbers of patients were different between the two groups because many patients were from different parts of the country and referred to our center only for second opinion and/or treatments, and many patients had no medical insurance and could not afford SSAs. Finally, the improvements in the complications prior to and following the treatment were not investigated. Further prospective controlled studies are required to elucidate whether individualized preoperative SSAs treatment can improve the surgical efficacy and provide evidence of the benefit of preoperative SSAs treatment in invasive macroadenoma patients.

## Conclusions

Currently, there is no consensus regarding the potential of preoperative SSAs treatment to improve the surgical remission rate in patients with acromegaly. Preoperative SSAs aiming to improve the surgical remission rate are not recommended by the Clinical Practice Guidelines of the Endocrine Society, the European Society of Endocrinology, and the Chinese Diagnostic and Treatment Guidelines for Acromegaly. Nevertheless, the present study suggests that preoperative SSAs improve the surgical remission rate in patients with invasive macroadenoma (Knosp grades 1–3) and that longer preoperative SSA treatment (˃6 months) improves the surgical results in specific cases. Consequently, the data suggest that the increase in the preoperative SSAs treatment duration, according to the disease conditions of the patients, has the potential to increase the surgical curative rate and improve the outcomes.

## Additional files

Additional file 1: Preoperative medical therapy data (XLSX 682 kb)

Additional file 2: Primary surgery data (XLSX 327 kb)

## Abbreviations

CCA: Cavernous carotid artery
CS: Cavernous sinus
GH: Growth hormone
IGF-1: Insulin-like growth factor 1
LAR: Long acting repeatable
MRI: Magnetic Resonance imaging
OGTT: Oral glucose tolerance test
SSAs: Somatostatin analogs
TSS: Transsphenoidal surgery
